# A path forward to improving the specificity of immunotherapies

**DOI:** 10.1002/ctm2.1051

**Published:** 2022-09-13

**Authors:** Brittany Barber, Florian Mair, Martin Prlic

**Affiliations:** ^1^ Department of Otolaryngology University of Washington Seattle Washington; ^2^ Department of Biology Institute of Molecular Health Sciences ETH Zurich Switzerland; ^3^ Vaccine and Infectious Disease Division Fred Hutchinson Cancer Center Seattle Washington United States; ^4^ Department of Global Health University of Washington Seattle Washington

Editorial

Despite impressive clinical successes, significant challenges persist in treatment of solid tumours with immune checkpoint inhibitor (ICI) therapy, including adverse effects and inability to reliably predict treatment response. Importantly, these challenges are not intrinsically linked to the underlying strategy of activating tumour‐specific T cells, but merely reflect that our current treatment methods are not selectively targeting intratumoural or tumour‐specific T cells. Such selective targeting of intratumoural immune processes has not been feasible in large part due to a gap in our knowledge about how these processes differ in inflamed and tumour tissues (Figure 1).

**FIGURE 1:**
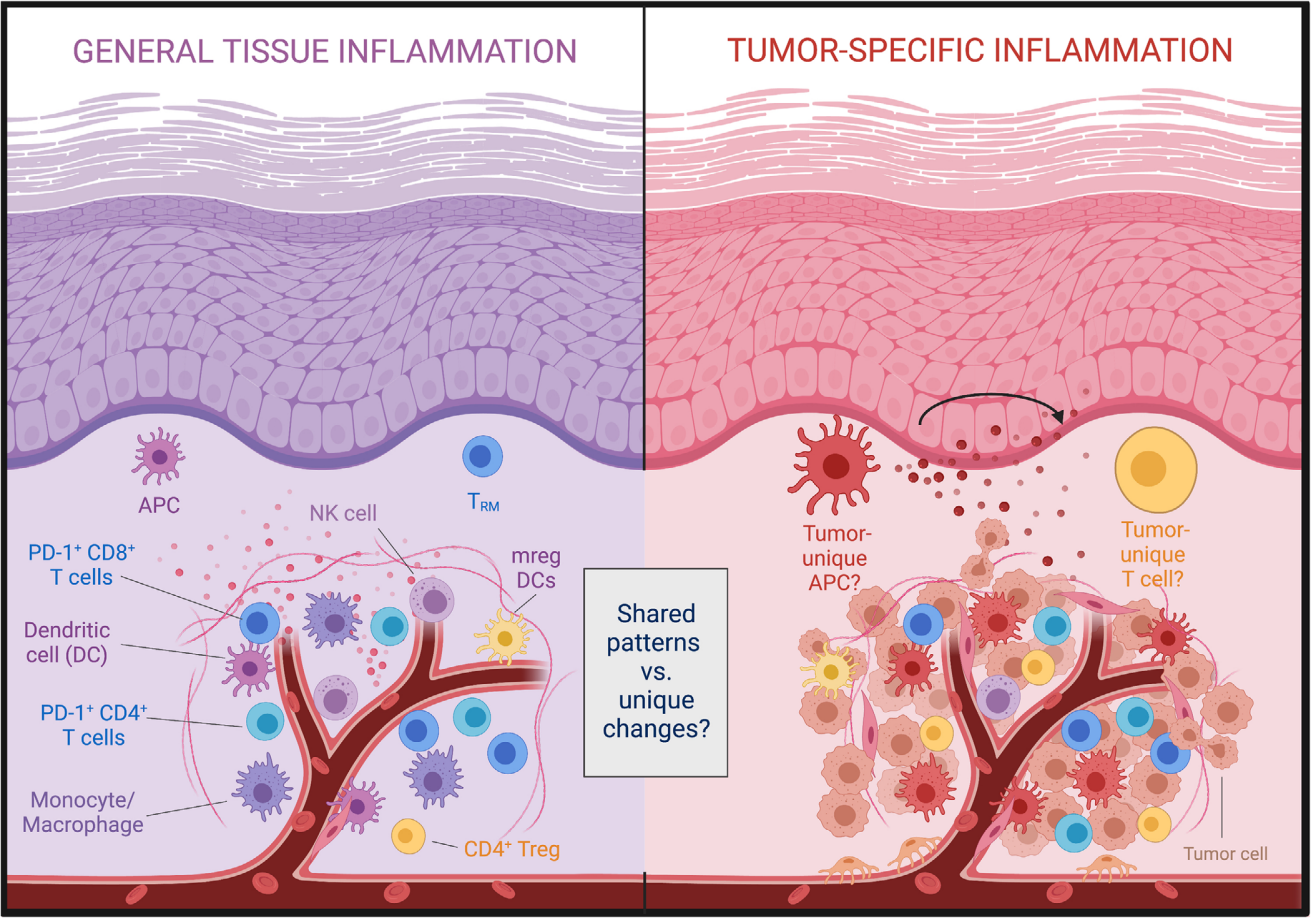
Identifying tumour‐specific immune changes and processes. While the immune infiltrate in tumours has been studied fairly extensively, little is known about immune processes in human inflamed tissues. We recently compared the immune infiltrate of inflamed (non‐malignant) oral mucosa and HNSCC tissues and found extensive immunological congruence including the presence of PD‐1+ expressing T cells as well as mature dendritic cells enriched in immunoregulatory molecules (mregDCs)

## 1 CURRENT TARGETS OF ICI ARE ALSO WIDELY EXPRESSED IN HEALTHY AND INFLAMED TISSUES

We recently compared the immune infiltrate in non‐malignant inflamed and tumour tissues using two different human cohorts – a cohort with inflamed oral mucosa (surgically discarded gingival tissue) and a cohort of both p16‐positive and p16‐negative head and neck squamous cell carcinoma (HNSCC) patients undergoing primary surgery.^1^ Overall, our study revealed that cell subsets and biomarkers often associated with the tumour microenvironment are also highly abundant in inflamed tissue. For example, the immune infiltrate in both tissues had an equal abundance of PD‐1‐expressing CD8 T cells.^1^ Of note, we observed in an earlier study that PD‐1‐expressing T cells were widely found even in healthy human oral mucosal tissues.^2^ These observations illustrate that cellular targets for the most common ICI treatment approach, antibodies against PD‐1 (pembrolizumab, nivolumab and cemiplimab) and PD‐L1 (atezolizumab, avelumab and durvalumab), are present in healthy and inflamed human tissues. This in turn raises the question if improving specificity is even possible. Are there immune processes of therapeutic relevance that are unique to or at least highly enriched in the tumour microenvironment?

## 2 DEFINING TUMOUR‐SPECIFIC IMMUNE CHANGES USING A COMBINATION OF SYNERGISTIC SINGLE‐CELL ANALYSIS METHODS

**FIGURE 2:**
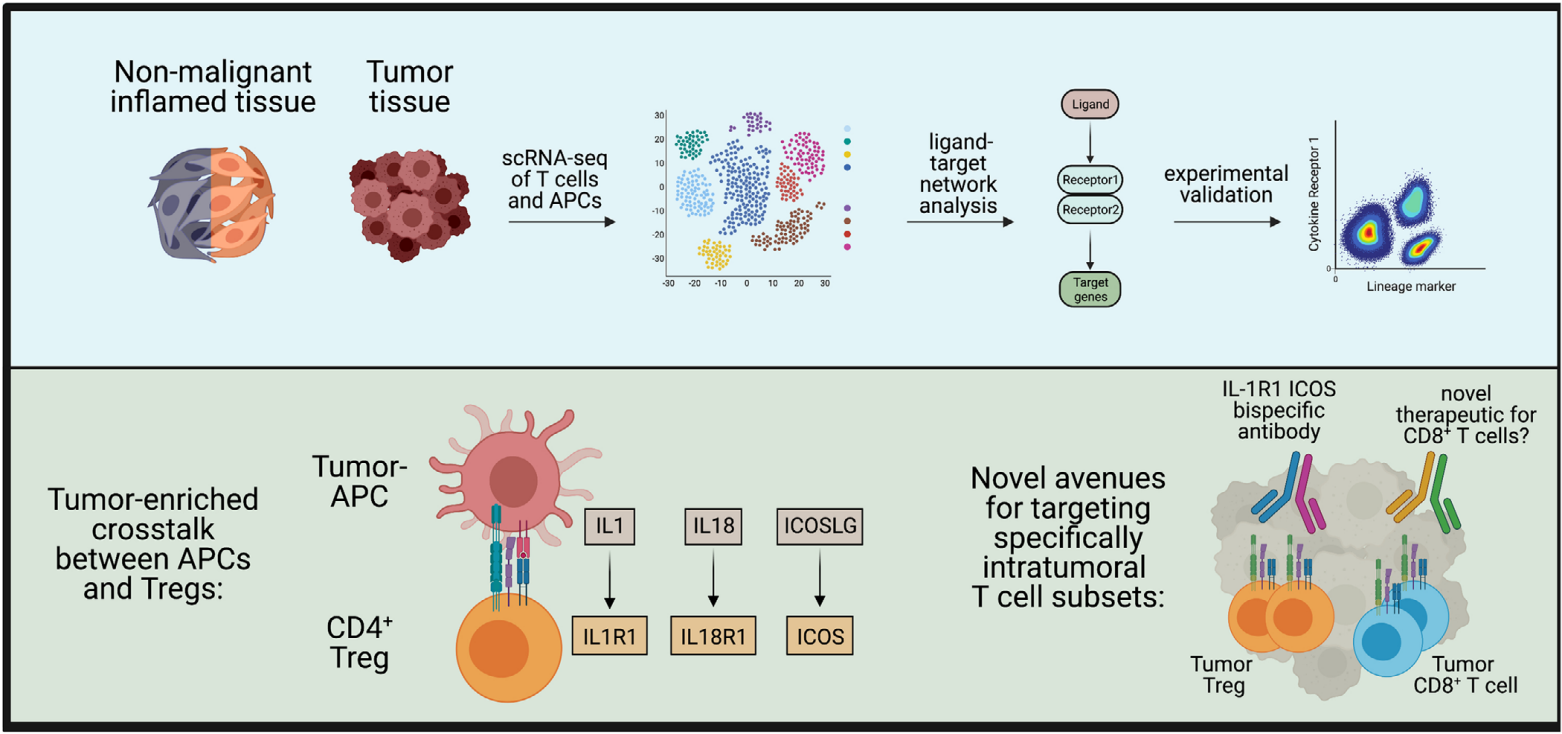
Discovering novel tumour‐specific immune cell interactions. We used NicheNet to predict which ligand‐receptor interactions between immune cells were enriched in HNSCC over inflamed tissues (blue box). We experimentally confirmed these predictions, which led to our discovery that IL1R1+ ICOS+ Tregs are highly enriched in HNSCC and induced by stimulation via their T‐cell receptor (green box, left). These Tregs could theoretically be depleted by a bispecific antibody to alter the immunosuppressive environment. If directly activating tumour‐specific CD8 T cells was also feasible, then this would allow for much more precise immunotherapies (green box, right)

In an effort to identify tumour‐unique immune processes, we applied novel machine learning approaches to our single‐cell data sets from inflamed oral mucosa and HNSCC tissues. We used ‘full annotation using shape‐constrained trees’ (FAUST),^3^ a machine learning algorithm that discovers and annotates statistically relevant cellular phenotypes in an unsupervised manner, to identify specific immune subset differences in our high parameter flow cytometry data. In parallel, we analysed our scRNA‐seq data sets with NicheNet,^4^ which is essentially a prediction tool for receptor‐ligand interactions that can be used to determine which interactions are enriched in one data set over another. Both approaches consistently identified ICOS‐expressing regulatory T cells (Tregs) as significantly enriched in the tumour relative to inflamed tissues. In addition, NicheNet predicted that intratumoural regulatory T cells receive IL‐1 signals via the IL‐1R1 receptor (Figure 2). We confirmed this prediction in follow‐up experiments and could demonstrate that the ICOS+ IL1R1+ regulatory T cells are massively expanded in HNSCC and also highly immunosuppressive.^1^ Importantly, no other haematopoietically derived cells in the blood or tumour co‐expressed ICOS and IL‐1R1. The identification of a phenotypically unique immune cell population in the tumour by just two proteins expressed on the cell surface now provides a promising new avenue for targeting these cells with bispecific antibodies and other approaches.

## 3 IS BROAD SPECTRUM ACTIVATION OF T CELLS THE ONLY OPTION FOR IMMUNOTHERAPIES?

Our study provides a path forward to targeting intratumoural Tregs for depletion in HNSCC, and possibly other solid tumours as well.^5^ Furthermore, it may shed light on the often variable treatment response observed between individual patients and solid tumour types (e.g. non‐small cell lung cancer vs. HNSCC). Such tumour‐specific depletion of Tregs could reduce the immunosuppressive environment of the tumour and eliminate, or at least minimise systemic side effects.

If targeting of intratumoural Tregs is feasible, could it also be possible to specifically target intratumoural CD8 T cells? Antibodies against PD‐1 or PD‐L1 were initially thought to disrupt the negative signals that PD‐1‐expressing T cells receive when PD‐1 interacts with its ligands PD‐L1, thereby rejuvenating exhausted T cells. However, it may actually also rely on additional indirect effects that induce pro‐inflammatory cytokines.^6^ It is noteworthy in this context that pro‐inflammatory cytokines are sufficient to induce expression of many biomarkers that are typically associated with T‐cell receptor‐mediated activation including PD‐1.^7^ These data further indicate that anti‐PD1 therapies might not merely affect exhausted, tumour‐specific T cells. Indeed, a recent study suggested that anti‐PD‐1 treatment impacts other concurrently occurring immune responses including responses to vaccination.^8^ Is there any indication that a more specific targeting approach of tumour‐specific T cells is feasible? A set of recent studies documented that the T‐cell infiltrate in human tumours can contain large populations of T cells that are not specific for tumour antigen, which can be phenotypically distinguished from tumour‐specific T cells.^9^, ^10^ These studies suggest that there is potential to identify and hence also therapeutically target intratumoural T cells in a more specific manner.

## 4 A PATH FORWARD TO IMPROVING THE SPECIFICITY OF IMMUNOTHERAPIES

Finally, our comparison of the immune infiltrate in inflamed oral and HNSCC tissues also highlights the feasibility of analysing human tissues to discover novel potential therapeutic targets. In the past, the field has typically relied on discoveries originating from the mouse model system to translate well to human disease. Similarly to immunotherapies, the mouse to human discovery approach has had great successes, but also has often failed. We hope that other groups will use either the data sets from our study (all of which are available in open access data repositories) or our general analysis strategy as a blueprint to discover more tumour‐ and disease‐specific targets by directly analysing human tissues, which would help ensure that potential therapeutic targets are of relevance. If discovery‐based approaches using human tissues continue at their current pace, more tumour‐ and pathology‐specific therapeutic targets should become available and find their way to the clinic. This increase in targeting specificity should go hand in hand with fewer toxic side effects and an improved ability to predict treatment response.

## DISCLOSURES

The experimental overview schemes in Figures 1 and 2 use templates from BioRender.com.
